# Factors associated with muscle mass in community-dwelling older people in Singapore: Findings from the SHIELD study

**DOI:** 10.1371/journal.pone.0223222

**Published:** 2019-10-09

**Authors:** Siew Ling Tey, Samuel Teong Huang Chew, Choon How How, Menaka Yalawar, Geraldine Baggs, Wai Leng Chow, Magdalin Cheong, Rebecca Hui San Ong, Farah Safdar Husain, Shuyi Charmaine Kwan, Cynthia Yan Ling Tan, Yen Ling Low, Ngiap Chuan Tan, Dieu Thi Thu Huynh

**Affiliations:** 1 Abbott Nutrition Research and Development, Asia-Pacific Center, Singapore; 2 Department of Geriatric Medicine, Changi General Hospital, Singapore; 3 Care and Health Integration, Changi General Hospital, Singapore; 4 SingHealth-Duke NUS Family Medicine Academic Clinical Program, Singapore; 5 Statistical Services, Cognizant Technologies Solution Pvt. Ltd., Bangalore, India; 6 Abbott Nutrition Research and Development, Columbus, Ohio, United States of America; 7 Health Services Research, Changi General Hospital, Singapore; 8 Department of Dietetic & Food Services, Changi General Hospital, Singapore; 9 SingHealth Polyclinics, Singapore; Ritsumeikan University, JAPAN

## Abstract

**Objectives:**

Aging is associated with low muscle mass and has been linked to adverse health outcomes. The objectives of this cross-sectional study were: (1) to describe anthropometry, body composition, appendicular skeletal muscle mass index (ASMI; appendicular skeletal muscle mass/height^2^), and prevalence of low ASMI in older people with normal nutritional status (Malnutrition Universal Screening Tool score = 0); (2) to determine factors associated with ASMI, and odds ratios of having low ASMI.

**Methods:**

SHIELD is a study of community-dwelling older people aged 65 years and above in Singapore. ASMI was determined using bioelectrical impedance analysis and low ASMI was defined as <7.0 kg/m^2^ for males and <5.7 kg/m^2^ for females (Asian Working Group for Sarcopenia, 2014).

**Results:**

A total of 400 older people (183 males and 217 females) took part in this study. The overall prevalence of low ASMI was 20.6% (15.5% in males and 24.9% in females). Females had significantly lower ASMI than males (*P* < 0.0001), age was inversely associated with ASMI (*P* = 0.0024) while BMI and calf circumference were positively associated with ASMI (both *P* < 0.0001) in the total cohort. In addition, ASMI was positively associated with bone mass in both genders (both *P* < 0.0001). After adjusting for covariates, the odds ratios of having low ASMI with every 1 year and 10 years increase in age were 1.13 (95% CI: 1.06, 1.20) and 3.36 (95% CI: 1.82, 6.21) respectively.

**Conclusions:**

The high prevalence of low ASMI in community-dwelling older people with normal nutritional status highlights the need for early screening. There was a strong inverse association between age and ASMI while BMI, calf circumference and bone mass were positively associated with ASMI. These findings will give further weight to the importance and development of public health strategies in maintaining and improving muscle health in this population group.

## Introduction

Approximately 10% of the world’s population will be aged 65 or older by 2025, and the proportion of older people population in Asia will grow from 6% in 2000 to 10% in 2025 [1]. One of the prominent changes associated with aging is the loss of skeletal muscle [2]. In 2019, the European Working Group on Sarcopenia in Older People (EWGSOP) revised consensus proposed three conceptual stages of sarcopenia, namely probable sarcopenia (low muscle strength), sarcopenia (low muscle strength plus low muscle quantity or quality) and severe sarcopenia (if all the above are present plus low physical performance) [3]. Low muscle mass is associated with adverse health outcomes such as functional impairment, physical disability, and morbidity in older people [4–8].

Appendicular skeletal muscle mass (ASM) is the sum of lean muscle mass from all four limbs [9, 10]. Appendicular muscle mass index (ASMI) is then derived from the ASM [4]. For the purpose of this paper, ASMI is defined as ASM divided by height squared (kg/m^2^). Based on the Asian Working Group for Sarcopenia (AWGS) recommendation, low muscle mass is characterized as ASMI <7.0 kg/m^2^ in males and <5.7 kg/m^2^ in females using bioelectrical impedance analysis (BIA) [10].

The literature suggests that age, gender and nutrition influence the decline in ASMI in older people. Seino et al. [11] performed a pooled analysis of four cohort studies in 4478 community-dwelling, non-disabled Japanese older people aged between 65 and 94 years. The prevalence of low ASMI (using BIA cut-off values defined by AWGS criteria) increased with age, ranging from 21.4% for 65–69 years to 73.1% for 85 years or older in men; and 29.9% for 65–69 years to 70.0% for 85 years or older in women [11]. Similar finding was reported in several Asian countries such as China [12], Japan [13, 14], Korea [15], Taiwan [16], Thailand [17], in which age was inversely associated with ASMI. Out of these studies, four cross-sectional studies examined this relationship in healthy community-dwelling adults aged ≥ 65 years in Japan and Korea [13–15], and ≥50 years in Taiwan [16]. These participants were recruited using random [15] and non-random [13, 14, 16] sampling methods. In addition, two cross-sectional studies examined the prevalence of sarcopenia in healthy adults in China (18–96 years of age who attended physical examination in the hospital) [12] and Thailand (20–84 years of age who were randomly selected from rural and urban areas) [17].

Although aging is an inevitable phenomenon, the difference in the rate of muscle mass loss between individual older people suggests the presence of modifiable factors that could potentially influence the loss of muscle mass [18]. For instance, previous research reported that skeletal muscle health was associated with modifiable factors such as physical activity and nutrition [3, 10, 18–22], anthropometry [23–26], BMI [12, 27–29], bone mineral density [12, 15, 29–31], and vitamin D status [32–36]. Malnutrition is also a known risk factor for low muscle mass, muscle strength, and physical function [3, 37].

A recent meta-analysis consisting of 113,967 older adults aged 60 and above reported that prevalence of community-dwelling older adults at risk of malnutrition is 26.5% (95% CI: 22.4%, 32.7%) [38]. However, it is unclear whether older people with normal nutritional status may also be at risk of sarcopenia.

To our knowledge, there is very limited data on muscle mass in community-dwelling older people with normal nutritional status. Factors associated with low muscle mass have not been well characterized in this population group. Understanding factors associated with low muscle mass can help to identify older people who are at risk of sarcopenia. This information can then be used to devise public health measures that may prevent or slow down the rate of muscle mass loss and its associated adverse health outcomes. Hence, community-dwelling older people with normal nutritional status were chosen as they may represent the ideal target for effective interventions before the onset of significant malnutrition and loss of muscle mass, leading to negative health outcomes.

The hypothesis of this study is that community-dwelling older people with normal nutritional status may be at risk of low ASMI. This risk is hypothesized to be lower as compared to the general population aged 65 years and above, as the general population would include older people at risk of malnutrition.

Thus, the objectives of this cross-sectional study were: (i) to describe anthropometry, body composition, ASMI, and prevalence of low ASMI in community-dwelling older people with normal nutritional status in Singapore; (ii) to examine factors associated with ASMI, and odds of having low ASMI.

## Methods

### Study design and participants

Strengthening Health In ELDerly through nutrition (SHIELD) is a study involving community-dwelling older people aged 65 years and above in Singapore. Participants in this cross-sectional study were recruited from the general public, community centers, polyclinics and hospital using non-random sampling through public talks, flyers, posters and referral from the healthcare professionals. To be eligible, participants were required to meet all the following inclusion criteria: Males or females participant aged ≥65 years, community ambulant with or without aid, Malnutrition Universal Screening Tool (MUST) score = 0. Participant was a community-dweller i.e. not staying in a residential intermediate, long-term care service institution or was being discharged home directly from hospital. In addition, participants with stable chronic disease(s), defined as any long-term medical condition on regular medications, with symptoms which did not vary beyond what was expected by each patient on a day-to-day basis when they were well, were also included. Participants were able to consume food and beverages orally, and able to communicate and follow instructions. The exclusion criteria were people with dementia, type 1 or type 2 diabetes, any active infectious disease (such as tuberculosis, Hepatitis B or C, HIV infection), severe gastrointestinal disorders (including but not limited to celiac disease, short bowel syndrome, pancreatic insufficiency), cystic fibrosis, end stage organ or pre-terminal diseases, acute myocardial infarction within the last 30 days from the screening, or active malignancy within the last five years.

The study was approved by the SingHealth Centralized Institutional Review Board in Singapore reference number 2017/2273. All participants provided written informed consent. The study was registered at clinicaltrials.gov as NCT03240952.

### Procedures

All study participants were asked to attend one visit at baseline, where participants’ socio-demographic information, co-morbidities, malnutrition status, blood samples, anthropometric measurements and body composition were collected. Functional assessment data on physical activity level and activities of daily living were also collected.

Socio-demographic data such as age, gender, ethnicity, marital status, education, number of prescribed drugs, smoker status, and alcohol consumption were collected during the visit. Participant’s comorbidity level was determined using Charlson Comorbidity Index (CCI) [39, 40]. Participant’s malnutrition status was performed using MUST to determine eligibility. MUST consists of three components, namely body mass index (BMI), weight loss, and acute disease that can affect risk of malnutrition due to non-oral intake [41].

Blood samples were obtained via venipuncture for the analyses of blood chemistry indicators associated with nutritional status such as 25-hydroxyvitamin D and creatinine. 25-hydroxyvitamin D was measured using immunochemistry analyzer COBAS e801 and vitamin D cut-off values were based on the definition described by Holick [42]. Creatinine was measured using chemistry analyzer COBAS c702 and estimated Glomerular Filtration Rate (eGFR) was then calculated using the Chronic Kidney Disease Epidemiology Collaboration (CKD-EPI) [43].

During the visit, anthropometric measurements and body composition were also collected. Standing height was measured without shoes by using a stadiometer (Avamech B1000) and the measurement was recorded to the nearest millimeter. Body weight and composition were measured to the nearest 0.1 kg using a bioelectrical impedance analyzer (Tanita MC-780) and were calibrated according to the manufacturer's specifications. Body Mass Index (BMI) was calculated by weight in kg divided by height squared in meter. BIA was used to estimate fat mass, muscle mass, and bone mass. Participants were weighed in light clothing without footwear. Mid upper arm circumference and calf circumference were measured with an anthropometric tape. Mid upper arm circumference was measured at mid-point of the acromion and olecranon. Calf circumference was measured at the largest part of the calf.

Participant’s physical activity level was determined using the Physical Activity Scale for the Elderly (PASE) [44, 45]. Modified Barthel Index (MBI) was used to measure functional independence for ten activities of daily living with dependency level score ranging from 0–20 as total dependent to 100 as independent [46].

### Data analysis

Baseline characteristics of the study participants were reported as means and standard error for continuous variables and numbers and percentages for categorical variables. For continuous variables, normality of the data was assessed using the Shapiro–Wilks test (*P* < 0.001) and graphical methods. Two sample *t* tests, chi-square tests and Wilcoxon rank sum test were used to compare the baseline characteristics, anthropometric measurements and body composition between females and males. Prevalence of low ASMI was determined based on the cut-off (BIA) recommended by the AWGS, i.e. males <7.0 kg/m^2^ and females <5.7 kg/m^2^ [10].

Stepwise multiple linear regression model was used to examine the associations between ASMI and all identified potential variables/factors. To select the candidate variable for stepwise multiple linear regression, the correlations between ASMI and each potential variable were determined using the Pearson correlation for continuous variables and t-tests or analysis of variance (ANOVA) for categorical variables. All variables with *P* < 0.10 from the correlation analysis were selected as candidate variables for inclusion in the stepwise regression models. Multicollinearity between the predictors were tested using variance inflation factor and tolerance. The highest variance inflation factor was 3.1 and the lowest tolerance was 0.32, which confirmed that there was no multicollinearity. Due to their relevance, stepwise selection were made with the forced inclusion of age and gender in the final gender-combined model and age in the by-gender models. Stepwise logistic regression model was used to examine the associations between low ASMI and potential predictors. Odds ratio and 95% CI were estimated from logistic regression models. Entry and stay criteria of alpha 0.05 was used in stepwise selection.

SAS version 9.4 (SAS Institute, Cary, NC, USA) was used for all statistical analyses. *P* < 0.05 was considered statistically significant.

## Results

Of the 415 community-dwelling older people initially screened for the study, 15 declined to take part and thus 400 older people, 183 males and 217 females, completed the study and were included in the analysis. **Table 1** shows characteristics of the total cohort, males (46%) and females (54%). Participants’ mean (SEM) age was 71.21 (0.26) years and predominantly Chinese (83.0%) with significantly higher percentage of Chinese in males compared to females (*P* = 0.0036). Majority of the participants were relatively healthy, with 372 (93.0%) participants classified as independent (having an MBI score of 100), and 392 (98.0%) participants having a Charlson Comorbidity score of 0. Only 2.5% were current smokers and 11% drank ≥ once per month. Over half of the study participants (52.0%) did not have sufficient vitamin D (<30 μg/L). Vitamin D concentration was significantly higher in males compared to females (*P* = 0.0084).

**Table 1:** Characteristics of the total cohort (*n* = 400), males (*n* = 183) and females (*n* = 217).

	Overall (*n* = 400)	Males (*n* = 183)	Females (*n* = 217)	*P* value (between genders)
Age (years)	71.21 (5.27)	71.26 (5.15)	71.18 (5.38)	0.8774
Age categories, *n* (%)				0.5939
<75 years	301 (75.2)	140 (76.5)	161 (74.2)	
≥75 years	99 (24.8)	43 (23.5)	56 (25.8)	
Ethnicity, *n* (%)				0.0036
Chinese	332 (83.0)	141 (77.0)	191 (88.0)	
Others	68 (17.0)	42 (23.0)	26 (12.0)	
Highest level of education, *n* (%)				0.0001
No formal education	62 (15.5)	18 (9.8)	44 (20.3)	
Secondary O/N level or equivalent	186 (46.5)	75 (41.0)	111 (51.2)	
A level or equivalent	100 (25.0)	60 (32.8)	40 (18.4)	
University and above	52 (13.0)	30 (16.4)	22 (10.1)	
Smoking status, *n* (%)				< 0.0001
Non-smoker	333 (83.25)	126 (68.9)	207 (95.4)	
Past smoker	57 (14.25)	48 (26.2)	9 (4.1)	
Daily/Occasional smoker	10 (2.50)	9 (4.9)	1 (0.5)	
Alcohol consumption in the last 12 months, *n* (%)				0.0034
No alcohol	287 (71.75)	119 (65.0)	168 (77.4)	
< Once a month	69 (17.25)	34 (18.6)	35 (16.1)	
≥ Once a month	44 (11.00)	30 (16.4)	14 (6.5)	
Physical Activity Scale for the Elderly score	119.45 (63.44)	122.86 (69.48)	116.58 (57.87)	0.3240
Modified Barthel Index score	99.54 (2.39)	99.43 (2.85)	99.64 (1.92)	0.3831
Modified Barthel Index, *n* (%)				
Moderate Dependence (60–79)	7 (1.8)	4 (2.2)	3 (1.4)	0.6737
Slight dependence (80–99)	21 (5.3)	11 (6.0)	10 (4.6)	
Independent (100)	372 (93.0)	168 (91.8)	204 (94.0)	
Total Charlson Comorbidity Score	0.03 (0.22)	0.04 (0.26)	0.02 (0.17)	0.3623
Charlson Comorbidity Score, *n* (%)				0.3757
0	392 (98.0)	178 (97.3)	214 (98.6)	
1	6 (1.5)	4 (2.2)	2 (0.9)	
2	1 (0.3)	0 (0.0)	1 (0.5)	
3	1 (0.3)	1 (0.5)	0 (0.0)	
25-hydroxyvitamin D (μg/L)	30.42 (10.14)	31.87 (10.66)	29.19 (9.53)	0.0084
25-hydroxyvitamin D, *n* (%)				0.0635
Deficient <20 μg/L	54 (13.5)	17 (9.3)	37 (17.1)	
Insufficient 20-<30 μg/L	154 (38.5)	71 (38.8)	83 (38.2)	
Sufficient 30–100 μg/L	192 (48.0)	95 (51.9)	97 (44.7)	
Height (cm)	158.68 (8.51)	165.57 (6.12)	152.87 (5.30)	< 0.0001
Body weight (kg)	61.86 (9.67)	66.75 (9.19)	57.74 (8.01)	< 0.0001
BMI (kg/m^2^)	24.53 (3.08)	24.31 (2.81)	24.71 (3.29)	0.1966
BMI categories, *n* (%)				0.0514
Normal weight (18.5–24.9 kg/m^2^)	245 (61.25)	121 (66.12)	124 (57.14)	
Overweight (25–29.9 kg/m^2^)	134 (33.50)	57 (31.15)	77 (35.48)	
Obese (≥30 kg/m^2^)	21 (5.25)	5 (2.73)	16 (7.37)	
Mid upper arm circumference (cm)	27.73 (3.27)	27.99 (2.99)	27.51 (3.49)	0.1474
Calf circumference (cm)	35.24 (3.21)	36.02 (3.15)	34.59 (3.11)	< 0.0001
Fat mass (kg)	17.87 (6.41)	14.79 (5.20)	20.43 (6.20)	< 0.0001
Fat (%)	28.50 (8.91)	21.60 (5.46)	34.25 (6.92)	< 0.0001
Muscle mass (kg)	41.57 (8.26)	49.14 (5.71)	35.25 (3.18)	< 0.0001
Muscle (%)	67.32 (8.40)	74.09 (5.18)	61.67 (6.08)	< 0.0001
Bone mass (kg)	2.38 (0.42)	2.71 (0.30)	2.11 (0.30)	< 0.0001
Appendicular skeletal muscle mass (kg)	17.75 (4.41)	21.67 (3.19)	14.48 (1.92)	< 0.0001
Appendicular skeletal muscle mass index (kg/m^2^)	6.96 (1.19)	7.88 (0.94)	6.19 (0.74)	< 0.0001
Low appendicular skeletal muscle mass index, *n* (%)				0.0207
Yes	82 (20.6)	28 (15.5)	54 (24.9)	
No	316 (79.4)	153 (84.5)	163 (75.1)	

All values are mean (standard deviation) unless otherwise specified.

Abbreviations: O/N level, General Certificate of Education: Ordinary Level / Normal Level; A level, General Certificate of Education: Advanced Level; BMI, body mass index.

Participants’ mean (SEM) BMI was 24.53 (0.15) kg/m^2^ and mid upper arm circumference was 27.73 (0.16) cm, with minimal differences in these measurements between females and males. Compared with females, males had higher calf circumference, weight, ASMI, and bone mass whereas fat mass was lower (all *P* < 0.0001). The overall prevalence of low ASMI was 20.6%, with females having higher prevalence of low ASMI than males (24.9% vs. 15.5%; *P* = 0.0207).

**Table 2** compares the characteristics between older people with normal ASMI (*n* = 316) and low ASMI (*n* = 82). Two participants did not have BIA data. When looking at the results of the total cohort, study participants with low ASMI were older, shorter, lighter, with lower BMI, mid upper arm circumference, calf circumference, fat mass, and bone mass than those with normal ASMI (all *P* < 0.0001). Similar results were found in male and female participants (all *P* < 0.0001).

**Table 2:** Characteristics of participants with normal ASMI and low ASMI.

	Overall (*n* = 398)			Males (*n* = 181)			Females (*n* = 217)		
	Normal ASMI (*n* = 316)	Low ASMI (*n* = 82)	*P* value	Normal ASMI (*n* = 153)	Low ASMI (*n* = 28)	*P* value	Normal ASMI (*n* = 163)	Low ASMI (*n* = 54)	*P* value
Appendicular skeletal muscle mass index (kg/m^2^)	7.27 (1.10)	5.77 (0.62)	<0.0001	8.13 (0.80)	6.52 (0.33)	<0.0001	6.47 (0.64)	5.37 (0.26)	<0.0001
Appendicular skeletal muscle mass (kg)	18.65 (4.30)	14.28 (2.88)	<0.0001	22.39 (2.84)	17.74 (1.92)	<0.0001	15.15 (1.67)	12.49 (1.09)	<0.0001
Age (years)	70.61 (4.83)	73.56 (6.25)	<0.0001	71.00 (5.16)	72.79 (5.06)	0.0933	70.25 (4.47)	73.96 (6.80)	<0.0001
Age categories, *n* (%)			<0.0001			0.1059			<0.0001
<75 years	253 (80.06)	46 (56.10)		120 (78.43)	18 (64.29)		133 (81.60)	28 (51.85)	
≥75 years	63 (19.94)	36 (43.90)		33 (21.57)	10 (35.71)		30 (18.40)	26 (48.15)	
Ethnicity, *n* (%)			0.7444			0.8066			0.4595
Chinese	263 (83.23)	67 (81.71)		118 (77.12)	21 (75.00)		145 (88.96)	46 (85.19)	
Others	53 (16.77)	15 (18.29)		35 (22.88)	7 (25.00)		18 (11.04)	8 (14.81)	
Highest level of education, *n* (%)			0.4972			0.3870			0.6261
No formal education	48 (15.19)	14 (17.07)		14 (9.15)	4 (14.29)		34 (20.86)	10 (18.52)	
Secondary O/N level or equivalent	152 (48.10)	33 (40.24)		66 (43.14)	8 (28.57)		86 (52.76)	25 (46.30)	
A level or equivalent	74 (23.42)	25 (30.49)		47 (30.72)	12 (42.86)		27 (16.56)	13 (24.07)	
University and above	42 (13.29)	10 (12.20)		26 (16.99)	4 (14.29)		16 (9.82)	6 (11.11)	
Smoking status, *n* (%)			0.6819			0.8939			0.7112
Non-smoker	262 (82.91)	70 (85.37)		106 (69.28)	19 (67.86)		156 (95.71)	51 (94.44)	
Past smoker	45 (14.24)	11 (13.41)		39 (25.49)	8 (28.57)		6 (3.68)	3 (5.56)	
Daily/Occasional smoker	9 (2.85)	1 (1.22)		8 (5.23)	1 (3.57)		1 (0.61)	0	
Alcohol consumption, *n* (%)			0.0875			0.1051			0.3595
No alcohol	226 (71.52)	60 (73.17)		101 (66.01)	17 (60.71)		125 (76.69)	43 (79.63)	
< once a month	60 (18.99)	9 (10.98)		31 (20.26)	3 (10.71)		29 (17.79)	6 (11.11)	
≥ once a month	30 (9.49)	13 (15.85)		21 (13.73)	8 (28.57)		9 (5.52)	5 (9.26)	
Physical Activity Scale for the Elderly score	122.08 (65.70)	108.91 (53.53)	0.0947	126.03 (71.65)	104.54 (55.97)	0.1342	118.36 (59.56)	111.19 (52.62)	0.4309
Modified Barthel Index score	99.48 (2.61)	99.74 (1.27)	0.3828	99.34 (3.11)	99.86 (0.52)	0.3816	99.62 (2.04)	99.69 (1.53)	0.8286
Total Charlson Comorbidity Score	0.03 (0.21)	0.02 (0.22)	0.9719	0.04 (0.28)	0.00 (0.00)	0.4577	0.01 (0.11)	0.04 (0.27)	0.3421
25-hydroxyvitamin D (μg/L)	30.62 (10.07)	29.82 (10.49)	0.5286	31.85 (10.43)	32.57 (12.16)	0.7459	29.45 (9.62)	28.40 (9.32)	0.4816
25-hydroxyvitamin D, *n* (%)			0.3693			0.5592			0.7544
Deficient <20 μg/L	39 (12.34)	15 (18.29)		13 (8.50)	4 (14.29)		26 (15.95)	11 (20.37)	
Insufficient 20-<30 μg/L	123 (38.92)	29 (35.37)		60 (39.22)	9 (32.14)		63 (38.65)	20 (37.04)	
Sufficient 30–100 μg/L	154 (48.73)	38 (46.34)		80 (52.29)	15 (53.57)		74 (45.40)	23 (42.59)	
Height (cm)	159.21 (8.48)	156.55 (8.44)	0.0116	165.78 (5.82)	164.68 (7.72)	0.3843	153.04 (5.38)	152.33 (5.04)	0.3947
Body weight (kg)	63.84 (9.31)	53.87 (6.09)	<0.0001	68.16 (8.79)	58.29 (6.16)	<0.0001	59.78 (7.86)	51.57 (4.64)	<0.0001
BMI (kg/m^2^)	25.16 (3.00)	21.97 (1.71)	<0.0001	24.77 (2.66)	21.46 (1.23)	<0.0001	25.53 (3.25)	22.23 (1.86)	<0.0001
BMI categories, *n* (%)			<0.0001			0.0003			<0.0001
Normal weight (18.5–24.9)	169 (53.48)	76 (92.68)		93 (60.78)	28 (100.00)		76 (46.63)	48 (88.89)	
Overweight (25–29.9)	127 (40.19)	6 (7.32)		56 (36.60)	0		71 (43.56)	6 (11.11)	
Obese (≥30)	20 (6.33)	0		4 (2.61)	0		16 (9.82)	0	
Mid upper arm circumference (cm)	28.33 (3.23)	25.40 (2.33)	<0.0001	28.45 (2.93)	25.45 (2.00)	<0.0001	28.22 (3.49)	25.38 (2.50)	<0.0001
Calf circumference (cm)	35.94 (2.99)	32.45 (2.36)	<0.0001	36.46 (3.07)	33.31 (2.05)	<0.0001	35.44 (2.84)	32.01 (2.40)	<0.0001
Fat mass (kg)	18.44 (6.77)	15.65 (4.12)	0.0004	15.08 (5.40)	13.23 (3.64)	0.0835	21.60 (6.41)	16.91 (3.80)	<0.0001
Fat (%)	28.36 (9.29)	29.03 (7.28)	0.5450	21.48 (5.48)	22.24 (5.43)	0.4988	34.82 (7.28)	32.55 (5.39)	0.0365
Muscle mass (kg)	42.97 (8.24)	36.15 (5.75)	<0.0001	50.33 (5.10)	42.67 (4.44)	<0.0001	36.07 (2.95)	32.78 (2.54)	<0.0001
Muscle (%)	67.39 (8.79)	67.07 (6.75)	0.7606	74.23 (5.21)	73.36 (5.02)	0.4170	60.96 (6.25)	63.81 (5.00)	0.0027
Bone mass (kg)	2.47 (0.40)	2.06 (0.36)	<0.0001	2.76 (0.27)	2.40 (0.29)	<0.0001	2.19 (0.28)	1.89 (0.25)	<0.0001

All values are means (standard deviation) unless otherwise specified.

Abbreviations: O/N level, General Certificate of Education: Ordinary Level / Normal Level; A level, General Certificate of Education: Advanced Level; BMI, body mass index.

A total of 16 potential variables/factors that may be associated with ASMI were identified based on the literature in this area and clinical relevance. All 16 variables were examined using univariate analysis (**Table 3**). Although BMI and mid upper arm circumference were strongly correlated with ASMI, BMI and mid upper arm circumference were highly correlated with each other (*r* = 0.6316, *P* < 0.0001). To improve the precision of the estimates and accuracy of the model, mid upper arm circumference was dropped from the model. There was a positive relationship between ASMI and BMI, mid upper arm circumference, calf circumference, bone mass, and creatinine (all *P* < 0.0001), and a tendency of a positive association between ASMI and PASE score (*P* = 0.0525). On the other hand, an inverse relationship was found between ASMI and age (*P* = 0.0274).

**Table 3:** Factors associated with ASMI using univariate analysis and ANOVA (*n* = 398).

	*P* value; *r* for continuous variables
Gender	*P* < 0.0001*
Ethnicity	*P* = 0.0252*
Education	*P* = 0.2377
Smoking status	*P* < 0.0001*
Alcohol consumption	*P* = 0.2540
Number of prescribed drugs currently taking	*P* = 0.0071*
eGFR (<60 mL/min/1.73 m^2^, ≥60 mL/min/1.73 m^2^)	*P* < 0.0001*
Age (years)	*P* = 0.0274; *r* = -0.1106*
Physical Activity Scale for the Elderly (PASE) score	*P* = 0.0525; *r* = 0.0973
Total Charlson Comorbidity Score	*P* = 0.8448; *r* = 0.0098
BMI (kg/m^2^)	*P* < 0.0001; *r* = 0.4398*
Mid upper arm circumference (cm)	*P* < 0.0001; *r* = 0.3724*
Calf circumference (cm)	*P* < 0.0001; *r* = 0.5611*
Bone mass (kg)	*P* < 0.0001; *r* = 0.7720*
25-hydroxyvitamin D (μg/L)	*P* = 0.1692; *r* = 0.0691
Creatinine (μmol/L)	*P* < 0.0001; *r* = 0.4694*

*P < 0.05. *r* is the Pearson correlation coefficient.

Abbreviations: eGFR, estimated Glomerular Filtration Rate; BMI, body mass index.

As shown in **Table 4**, factors that were associated with ASMI include gender, age, BMI, and calf circumference in the total cohort. Females had lower ASMI than males (*P* < 0.0001). In the total cohort, every 1-year increase in age holding other factors constant, ASMI was significantly lower by 0.017 kg/m^2^ (95% CI: 0.006, 0.027, *P* = 0.0024) and ASMI was positively correlated to BMI and calf circumference (both *P* < 0.0001). When examining gender-specific models, ASMI was positively correlated to calf circumference (*P* < 0.0001) and PASE score in males (*P* = 0.0052). ASMI was positively associated with BMI and bone mass in both genders (all *P* < 0.0001).

**Table 4:** Factors associated with ASMI using multiple linear regression models.

	Total (*n* = 398)			Males (*n* = 181)			Females (*n* = 217)		
	β	95% CI	*P* value	β	95% CI	*P* value	β	95% CI	*P* value
Gender									
Males	0								
Females	-1.663	-1.780, -1.545	< 0.0001						
Age (years)	-0.017	-0.027, -0.006	0.0024	-0.010	-0.025, 0.005	0.1866	-0.010	-0.023, 0.003	0.1409
BMI (kg/m^2^)	0.150	0.128, 0.173	< 0.0001	0.175	0.139, 0.211	< 0.0001	0.133	0.110, 0.155	< 0.0001
Calf circumference (cm)	0.069	0.047, 0.091	< 0.0001	0.065	0.033, 0.096	< 0.0001			
Bone mass (kg)				0.723	0.402, 1.043	< 0.0001	0.604	0.356, 0.852	< 0.0001
PASE score				0.002	0.0005, 0.003	0.0052			

Abbreviation: BMI, body mass index; PASE, physical activity score for the elderly.

A total of 16 variables were examined using univariate logistic regression analysis (**Table 5**) and 7 variables were found to be significantly associated with low ASMI (all *P* < 0.05). BMI and mid upper arm circumference were significantly highly correlated with each other, only BMI was included in the stepwise multiple logistic regression model together with other variables that were associated with low ASMI (*P* < 0.10). Females had significantly higher odds of having low ASMI than males (*P* = 0.0218). With every 1-year increase in age, the odds of having low ASMI increased by 10% (Odds ratio = 1.10, 95% CI: 1.05, 1.15). Participants with higher BMI, mid upper arm circumference, calf circumference, bone mass, and creatinine had lower odds of having low ASMI (all *P* ≤ 0.0353).

**Table 5:** Factors associated with low ASMI using logistic regression models (*n* = 398).

	OR	95% CI	*P* value
Gender			0.0218*
Males (ref)	1.00		
Females	1.81	1.09, 3.01	
Ethnicity			0.7445
Chinese (ref)	1.00		
Others	1.11	0.59, 2.09	
Education			0.5002
No formal education/primary (ref)	1.00		
Secondary or O/N level or equivalent	0.74	0.37, 1.51	
A level or equivalent	1.16	0.55, 2.45	
University and above	0.82	0.33, 2.03	
Smoking status			0.6961
Non-smoker (ref)	1.00		
Past smoker	0.92	0.45, 1.86	
Daily/Occasional smoker	0.42	0.05, 3.34	
Alcohol consumption			0.0940
No alcohol (ref)	1.00		
< once a month	0.57	0.27, 1.20	
≥ once a month	1.63	0.80, 3.32	
Number of prescribed drugs currently taking			0.7716
Nil (0) (ref)	1.00		
One to five (1–5)	0.92	0.50 1.69	
More than five (>5)	1.15	0.55, 2.42	
eGFR			0.6329
Mildly decreased/Normal ≥60 (ref)	1.00		
Moderately/Severely decreased <60	0.84	0.40, 1.74	
Age (years)	1.10	1.05, 1.15	< 0.0001*
Physical Activity Scale for the Elderly score	0.99	0.99, 1.00	0.0957
Total Charlson Comorbidity Score	0.98	0.31, 3.14	0.9721
BMI (kg/m^2^)	0.50	0.42, 0.60	< 0.0001*
Mid upper arm circumference (cm)	0.70	0.63, 0.78	< 0.0001*
Calf circumference (cm)	0.64	0.57, 0.72	< 0.0001*
Bone mass (kg)	0.06	0.03, 0.12	< 0.0001*
25-hydroxyvitamin D (μg/L)	0.99	0.97, 1.02	0.5277
Creatinine (μmol/L)	0.99	0.98, 1.00	0.0353*

*P < 0.05

Abbreviations: eGFR, estimated Glomerular Filtration Rate; BMI, body mass index.

In univariate analysis, we observed that females had higher odds of low ASMI than males (**Table 5**). However, in the presence of bone mass in the multiple logistic regression model, we saw an unexpected reversal in the odds, where females showed lower odds of having low ASMI. We found the reversal to be a case of Simpson’s Paradox [47], most often a consequence of an imbalance in the sample size at the levels of the confounding variable. In this study, the higher numbers of males than females with higher bone mass and lower numbers of males than females with lower bone mass created the reversal in relationship between gender and ASMI. The plot (**S1 Fig**) illustrates the reversal of odds of females having low ASMI (<5.7 kg/m^2^) vs. males in the presence of bone mass. In the first tertile of bone mass (1.3–2.1 kg), the percent of males with low ASMI (<7.0 kg/m^2^) is 100% vs. 36.52% in females. In the second tertile of bone mass (2.2–2.5 kg), 34.04% vs. 13.95% of males vs. females have low ASMI. Finally, in the third tertile of bone mass (2.6–3.7 kg), 5.43% vs. 0% of males vs. females have low ASMI. Thus, we decided to fit bone mass separately within genders, but exclude bone mass from the model with combined genders.

There was an association between low ASMI and gender, age, BMI, and calf circumference in the total cohort (**Table 6**). Females had higher odds of having low muscle mass compared to males and the odds of having low muscle mass increased significantly with age but decreased significantly with higher BMI and calf circumference in the total cohort (all *P* ≤ 0.0261). When looking at gender-specific models, low ASMI was associated with BMI and bone mass for both genders (all *P* ≤ 0.0080). In addition, low ASMI was associated with age and calf circumference in females (both *P* ≤ 0.0415).

**Table 6:** Factors associated with low ASMI using multiple logistic regression models (*n* = 398).

	Total (*n* = 398)			Males (*n* = 181)			Females (*n* = 217)		
	OR	95% CI	*P* value	OR	95% CI	*P* value	OR	95% CI	*P* value
Gender									
Males (ref)	1								
Females	2.10	1.09, 4.04	0.0261						
Age (years)	1.13	1.06, 1.20	0.0001	1.05	0.94, 1.18	0.3799	1.14	1.05, 1.24	0.0020
BMI (kg/m^2^)	0.55	0.45, 0.67	< 0.0001	0.39	0.25, 0.62	< 0.0001	0.63	0.50, 0.79	< 0.0001
Calf circumference	0.80	0.70, 0.91	0.0005				0.83	0.69, 0.99	0.0415
Bone mass (kg)				0.03	0.00, 0.41	0.0080	0.04	0.01, 0.23	0.0006

Abbreviation: BMI, body mass index.

**Fig 1:**
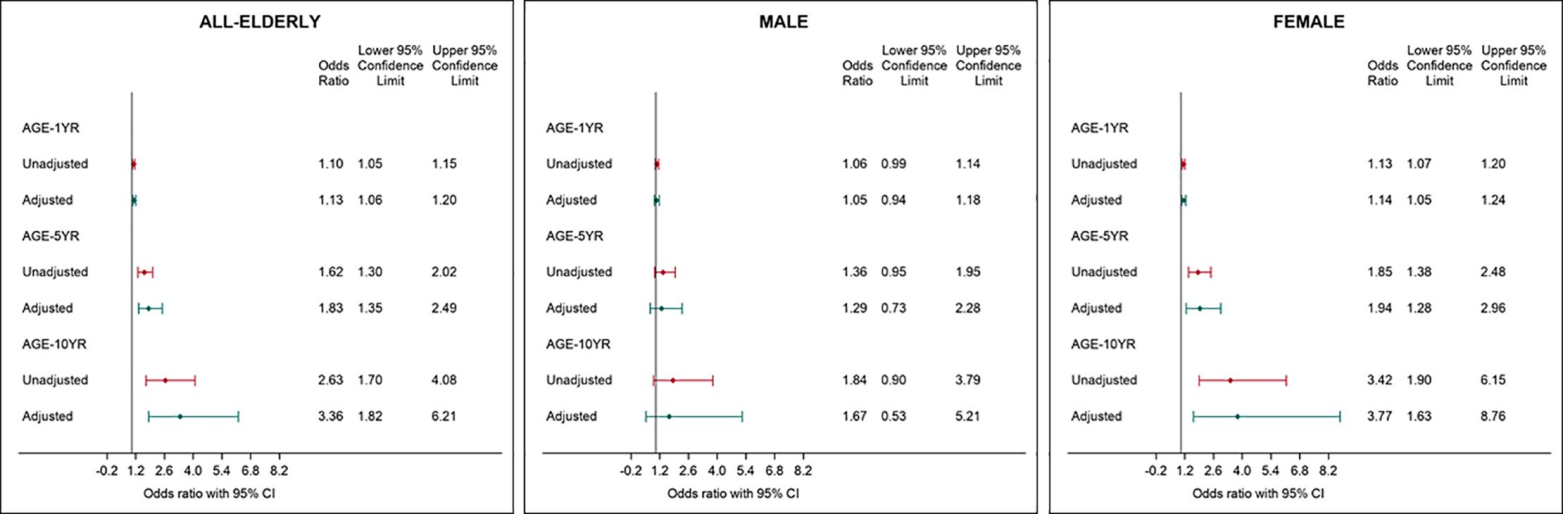
Association between low ASMI and age increment (1 year, 5 years, 10 years) using logistic regression models in (A) all older people* (*n* = 398), (B) Males† (*n* = 181), and (C) Females‡ (*n* = 217). A. *Adjusted for gender, BMI and calf circumference. B. †Adjusted for BMI and bone mass. C. ‡Adjusted for BMI, calf circumference and bone mass.

**Fig 1** shows the unadjusted and adjusted odds ratio (95% CI) for low ASMI with 1 year, 5 years, and 10 years increased in age for all older adults, males, and females. With every 1 year, 5 years, and 10 years increase in age, the unadjusted odds ratio of having low ASMI increased by 1.10, 1.62, and 2.63 times respectively, and the adjusted odds ratio was 1.13, 1.83, and 3.36 times respectively (all *P* < 0.05). After adjusting for covariates, females had 3.77 (95% CI: 1.63, 8.76) times higher odds of having low ASMI with every 10 years increase in age.

## Discussion

To our knowledge, this is the first study of its kind to determine the prevalence of ASMI and factors associated with ASMI in independently ambulant community-dwelling older people, aged 65 years and above with normal nutritional status in Singapore. The overall prevalence of low ASMI using AWGS definition for BIA was 20.6%. Results from multiple linear regression models showed that ASMI was positively correlated to gender (males), BMI and calf circumference in the total cohort, as well as BMI and bone mass in the gender-specific models. Age is an important risk factor of low ASMI. Similar findings were found in the multiple logistic regression models. After adjusting for gender, BMI, and calf circumference, every 1-year increase in age was associated with 13% higher odds of having low ASMI and every 10 years increase in age was associated with 3.4-fold greater odds of low ASMI.

The older people in the present study were relatively healthy with high MBI score and low Charlson Comorbidity score. They had a higher level of education as compared with the general population aged 65 years and above in Singapore [48]. In spite of the above, a high prevalence of low ASMI (15.5% males, 24.9% females) was still observed.

The present study found that ASM and ASMI were lower in females than males, and the prevalence of low ASMI was higher in females (24.9%) than in males (15.5%). The relationship persists even after adjusting for potential confounders. A few studies reported gender-related differences in muscle mass, where males were found to have significantly higher ASM and ASMI than females [14, 16, 34, 49, 50]. Recent cross-sectional studies in adults aged 60 years and above reported that the prevalence of low ASMI using AWGS criteria for BIA was 19.6%-26.5% in males and 19.7%-31.9% in females [29, 51]. Further to this, two studies in community-dwelling Japanese older adults used EWGSOP criteria (men <7.0 kg/m^2^ and women <5.8 kg/m^2^) [52] reported that prevalence of low ASMI was 32.2%-38.4% in males and 38.7%-48.9% in females [53, 54]. These two studies did not exclude malnourished older adults. When all the results are taken together, it appears that the prevalence of low ASMI observed in the current study tends to be lower than the prevalence reported in other Asian countries, which included both nourished and malnourished participants. The difference in nutritional status of the participants between the current study and previous studies is the likely explanation for this observation.

One of the novel aspects of the study is that we investigated the prevalence of low ASMI in independently ambulant community-dwelling older people with normal nutritional status using MUST tool. Recent studies reported that malnutrition is a risk factor for low muscle mass, muscle strength, and physical function [3, 37]. In Singapore, prevalence of moderate or high nutritional risk using DETERMINE (score greater than 3) was 30.1% among community-dwelling older people aged 55 years and above [55]. As such, the prevalence of low ASMI in the general population aged 65 years and above in Singapore is likely going to be higher than the prevalence observed in the present study, as they would include individuals who are at risk of malnutrition. The present study extends our knowledge from current literature by showing that one in five older people with normal nutritional status will potentially be at risk of sarcopenia. This highlights the importance of early screening for sarcopenia even among well-nourished, independently ambulant, community-dwelling older adults in Singapore.

A further finding of our study was the inverse relationship between age and ASMI. Among the older adults aged 65 to <75 years, the prevalence of low ASMI for males was 13.0% and females was 17.4%, whereas for those aged ≥75 years, the prevalence for males was 23.3% and females was 46.4%. This is in line with previous research in other Asian countries such as China [12, 50], Japan [11, 13, 14, 49, 53], Korea [15], Taiwan [16], Thailand [17], where age was associated with low muscle mass. Further to this, Auyeung et al. quantified the loss of muscle mass in a longitudinal observational study in 3018 Chinese older people. During a 4-year follow-up period, an ASM loss of −1.59% in men and −2.02% in women was observed [56].

There is growing evidence that calf circumference can be used as an accurate indicator of ASM and nutritional status [23–26]. We found a positive association between ASMI and calf circumference. This finding is in line with two recent studies in community-dwelling older adults aged 65 years and above, in which a positive relationship was found between ASMI and calf circumference, ranging from 0.54 to 0.78 in males and 0.42 to 0.75 in females [24, 54]. Kawakami et al. [23] suggested the cut-off values for calf circumference to predict low ASMI (6.87 kg/m^2^ in males and 5.46 kg/m^2^ in females) were <34.1 cm in males and <32.8 cm in females. In addition, Kim et al. [24] reported cut-off values to predict reduced muscle mass were 35 cm for males and 33 cm for females. These cut-off values were very close to the calf circumference values observed in the present study, i.e. 33.3 cm for males with low ASMI and 32.0 cm for females with low ASMI. Further to this, the 2019 revised European consensus on sarcopenia recommends the use of calf circumference as a surrogate measure for older adults when other muscle mass assessment methods are not available [3]. Overall, calf circumference seems to be a viable alternative measurement to assess for low ASMI based on our study findings. It has the added advantage of being easily administered in the community as a screening instrument.

Our study demonstrated that older people with normal ASMI had higher BMI than those with low ASMI. Several recent studies conducted in Asian older adults aged 60 years and above reported a significant positive association between BMI and ASMI, and BMI was inversely associated with the odds of having low ASMI [29, 54, 57, 58]. Some studies have suggested that higher BMI is associated with better health outcomes in the older population [59–61]. However, sarcopenic obesity would be detrimental to health outcomes in the older population [62]. This highlights the importance of assessing muscle health in addition to BMI [63, 64].

Older people with normal ASMI in the present study had higher bone mass compared with those with low ASMI. A few cross-sectional studies with large sample size (>3000 Asian older adults) have shown a positive relationship between ASMI and bone mineral density in different sites [15, 30, 57]. Recent reviews reported that the loss of muscle is accompanied by the loss of bone mass during aging [65, 66]. There is growing evidence that muscle and bone interact with each other via cross talk, and there are biomechanical and biochemical links between muscle and bone [67]. A cross-sectional study reported that older males in lower ASMI group (ASMI <6.85 kg/m^2^) had 2.2 times higher odds of having osteoporosis, even after adjusting for osteoporotic risk factors such as low bone mineral density [30]. Given the strong muscle and bone relationship, public health strategies which target these two factors singly or in unison, could potentially counteract the loss of both muscle mass and bone mass. This may lead to a delay or prevention of sarcopenia and osteoporosis simultaneously.

While aging is an inevitable phenomenon, modifiable factors such as nutrition, physical activity and lifestyle have been shown to positively influence muscle health. Several recent reviews and consensus statements have consistently demonstrated beneficial effects of physical activity and nutritional interventions (e.g. protein, β-hydroxy-β-methylbutyrate (HMB), vitamin D) on muscle health [3, 10, 19, 63, 64, 68–81] and bone health [82–86] in older people. In particular, resistance exercise training, high protein diet, and HMB supplementation have been shown to prevent or reverse sarcopenia in older persons [63, 64, 71–80]. We found a positive association between PASE and ASMI in males. This finding is in agreement with results from previous studies which reported a beneficial relationship between physical activity and muscle mass in older adults [21, 22, 87] in a dose-response manner, in which elderly with moderate or vigorous physical activity ≥150 min per week has greatest amount of muscle mass [88]. Thus, it is important to promote physical activity and diet quality among older people for optimal muscle and bone health.

Our study found that 52% of the community-dwelling older people had either vitamin D deficiency with serum 25(OH)D concentration <20 μg/L (males: 9.3%, females: 17.1%) or insufficiency with serum 25(OH)D concentration 20 to <30 μg/L (males: 17.1%, females: 38.2%). Similar prevalence of vitamin D deficiency and insufficiency has been previously reported in Asian elderly aged ≥60 years [36, 89, 90]. Thus, the high prevalence of suboptimal vitamin D status in this study among older adults with normal nutritional status, residing in a sunny tropic region, is concerning. This reinforces the need to heighten the awareness among clinicians to encourage screening and monitoring of vitamin D status in older adults. Sun exposure and vitamin D rich foods and supplementation are means to improve vitamin D status in older people [89–91].

The present study found no evidence of an association between serum vitamin D and ASMI. Previous research in this area is somewhat mixed. Studies with large sample size showed that there was a positive relationship between vitamin D concentration and muscle mass [33, 34]. One of these studies reported that male participants suffering from hypovitaminosis defined as serum 25(OH)D below 20 ng/mL (same as 20 μg/L) had a lower ASMI than their counterparts with normal vitamin D levels [34]. In contrast, a cross-sectional study comprising of 912 Chinese elderly reported a positive association between vitamin D concentration and grip strength, but not muscle mass, in males [36]. It appears that the association between vitamin D and muscle mass becomes more apparent in older people with vitamin D deficiency. It is possible that the present study did not have sufficient power to detect an association between vitamin D and ASMI.

To the best of our knowledge, this is the first study of its kind to examine muscle mass in independently ambulant community-dwelling older people with normal nutritional status in Singapore. The strengths of this study include measurements of a wide range of variables such as socio-demographic and lifestyle habits, anthropometric measurements, and biochemical indices to account for potential determinants of ASMI. In addition, the use of BIA provided a safe, convenient, affordable and practical way to measure ASM in this study. Several studies have previously reported on the validity of measuring ASM using BIA [92, 93]. Although dual energy X-ray absorptiometry (DXA) has been recommended as the gold standard for ASM measurement, it does not have the above-mentioned advantages of BIA [9, 63]. Further studies are warranted to confirm the reliability, validity, and reproducibility of using BIA to measure ASMI in older people.

This study was not without limitations. One limitation is that this study was conducted in older people who were relatively healthy using non-random sampling, and thus generalization of results to other population groups should be done with caution. Although there are limitations associated with the common use of stepwise regression analysis, the factors identified in this study have been shown in previous studies to have significant associations with low ASMI. In addition, separate models for males alone, females alone, and genders combined share common significant predictors pointing to some level of model validation. In addition, the cross-sectional nature of the study means that causal relationship between low ASMI and factors associated with it could not be established. Future prospective studies are needed to confirm the associations in this study. Further research is warranted to elucidate the underlying mechanisms and the potential role of lifestyle interventions in improving muscle health in older adults.

In conclusion, one in five community-dwelling older people with normal nutritional status had low ASMI. Females (24.9%) were more affected than males (15.5%). In addition, age is an important risk factor of low ASMI. Every 10 years increase in age was associated with 3.4-fold greater odds of low ASMI. On the other hand, BMI, calf circumference and bone mass were positively associated with ASMI. These findings could be used to identify older adults who are at risk of low ASMI and devise effective public health strategies to prevent or delay the progression to sarcopenia in this population group.

## Supporting information

S1 Fig Relationship between bone mass and ASMI in males and females. (TIFF)
