# Supplementary figures and images for: Factors associated with muscle mass in community-dwelling older people in Singapore: Findings from the SHIELD study
Source: PLoS One. 2019 Oct 9;14(10):e0223222. doi: 10.1371/journal.pone.0223222 (PMC6785067; doi:10.1371/journal.pone.0223222)

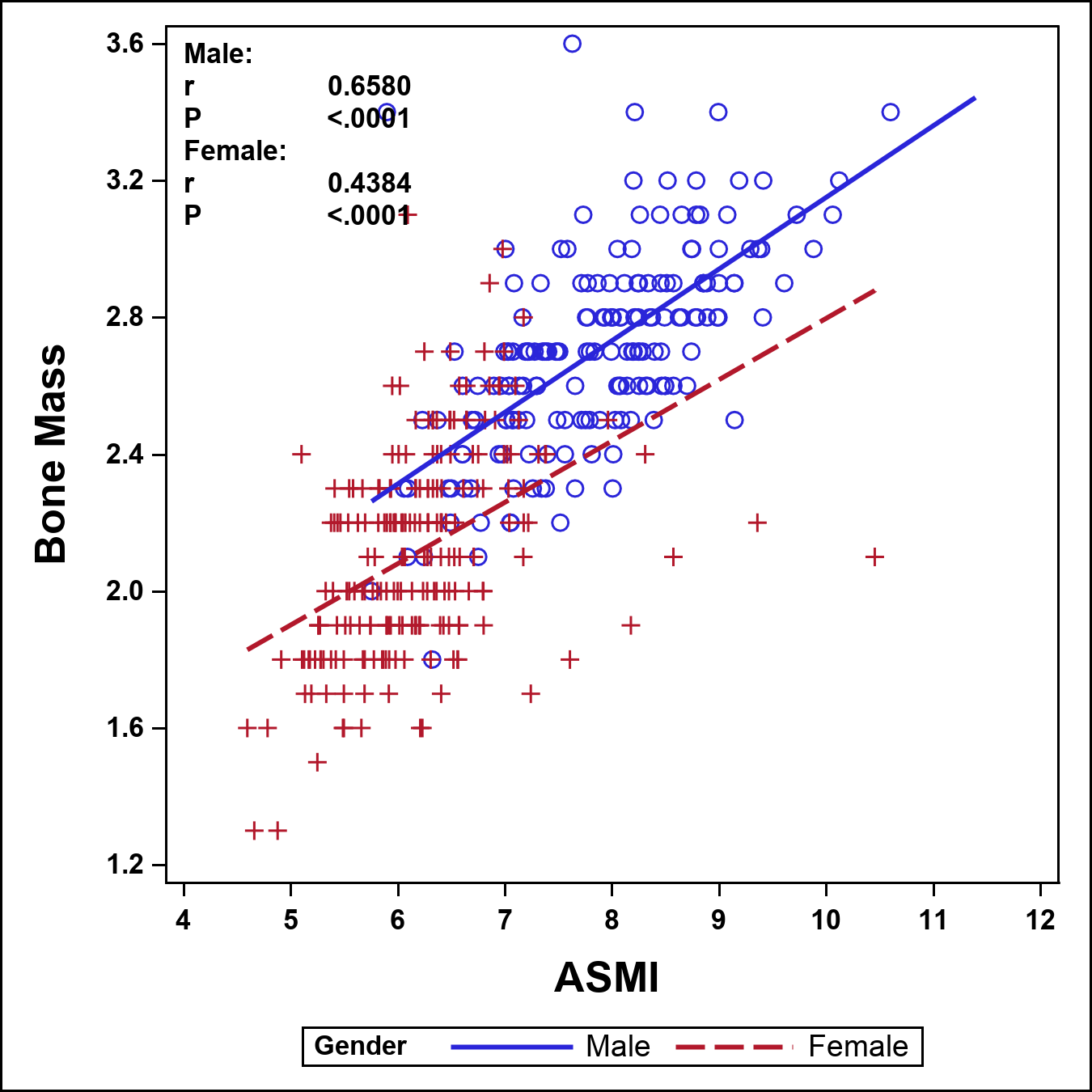

Supplement: S1 Fig — (TIFF) [file pone.0223222.s001.tiff]
